# The Metabolic Profile of Long-Lived *Drosophila melanogaster*

**DOI:** 10.1371/journal.pone.0047461

**Published:** 2012-10-23

**Authors:** Pernille Sarup, Simon Metz Mariendal Pedersen, Niels Chr. Nielsen, Anders Malmendal, Volker Loeschcke

**Affiliations:** 1 Department of Bioscience, Integrative Ecology and Evolution, Aarhus University, Aarhus, Denmark; 2 Center for Insoluble Protein Structures, Aarhus University, Aarhus, Denmark; 3 Department of Food Science, Aarhus University, Tjele, Denmark; 4 Department of Biomedical Sciences, University of Copenhagen, Copenhagen, Denmark; Lancaster University, United Kingdom

## Abstract

We investigated the age-related changes in the metabolic profile of male *Drosophila melanogaster* and compared the metabolic profile of flies selected for increased longevity to that of control flies of equal age. We found clear differences in metabolite composition between selection regimes and among age groups. Contrary to results found in a previous study of the transcriptome of these lines the metabolic profile did not show a younger pattern in longevity-selected (LS) flies than in same aged control (C) flies. Rather, many of the metabolites affected by age had levels common to older control individuals in the young LS flies. Furthermore, ageing affected the metabolome in a different LS specific direction. The selection induced difference increased with age. Some metabolites involved in oxidative phosphorylation changed with age highlighting the importance of mitochondrial function in the ageing process. However, these metabolites were not affected by selection for increased longevity, indicating that improvements of mitochondrial function were not involved in the increased lifespan of LS lines. Of the eight metabolites identified as having a significant difference in relative abundance between selection regimes in our study choline, lysine and glucose also show difference among lifespan phenotypes in *C. elegans* indicating that the correlation between the concentration of these metabolites and longevity was evolutionary conserved. Links between longevity and choline concentration is also found in mice making this metabolite an obvious target for further study.

## Introduction

Due to vast improvements in nutrition, housing and medicine during the last century we have seen a dramatic increase in the average expected lifespan of humans in the developed part of the world. This fact combined with a decrease in birthrate has led to a growing proportion of elderly and emergence of age as the major risk factor for the most frequent diseases and causes of death in industrialized countries, including cancer, cardiovascular defects, diabetes and dementia.

Even though our primary interest is to understand, and perhaps modulate, the rate or onset of aging in humans [1], [2], we need to look first at more basic animal models, which can provide faster and some times more accurate answers. Model organisms such as fruitflies (*Drosophila melanogaster*), worms (*Caenorhabditis elegans*) and mice (*Mus musculus*) share many of the common features of complex systems of metazoan cells with humans. As aging insect tissues show many of the structural changes that occur in mammals, e.g. deposition of lipofuscin or degenerated mitochondria [3], gerontology studies using insect models are not solely of academic interest. They help us to elucidate the interaction between environment, genome and phenotype in metazoan organisms, and could thus help us to identify mechanisms that also influence aging in humans [4].

We have chosen to work with the model organism *D. melanogaster*. The comparably short lifespan of *D. melanogaster* [5] allows us to select for increased longevity in numerous generations, and thereby to obtain a remarkable response. Generally, there are two ways to select for increased longevity in *Drosophila*: selection on virgin lifespan [6] or mated lifespan [7]. Evidence suggests that the genetic determination of lifespan of mated and virgin flies is at least partly different. The selection responses after selection for virgin longevity have been reported to disappear when mated lifespan is measured [8]. After selection on mated lifespan the selection response can be retained in virgin flies [9]. Following evolutionary theories [10]–[13], senescence should not take its toll on the organism before reproduction is initiated. Selection on increased mated lifespan by only allowing successful reproduction to take place after 50% of the cohorts have died should therefore in theory postpone the onset of senescence. This seems to have been the outcome of a selection program in our laboratory [14]. The resulting longevity selected (LS) lines have an 66% increased median lifespan compared to control (C) lines, where flies were allowed to reproduce within a week after hatching. In a genome-wide investigation of the differences in gene-expression profile between LS and C lines in young, middle-aged and old male flies we identified the genes that were significantly differently expressed between LS and C lines [14]. Using expression profiles of these genes in a hierarchical clustering LS flies consistently clustered with C flies that were one age class younger. The young gene expression profile in LS lines indicates that LS flies with regards to part of the transcriptome are physiologically younger than same aged C flies. To test this hypothesis of a younger phenotype in aging LS lines compared to same aged C lines further on a different level of biological organization we used ^1^H-NMR metabolomics to characterize the global metabolome in samples of whole *D. melanogaster*. This method has successfully been used before to identify metabolites of interest in *D. melanogaster* and other organisms [15]–[21], and also for studies of ageing [22]. Ageing results in changes and decline of function on genetic, transcriptomic and translational levels all of which potentially will result in changes in the metabolome. Investigation of the ageing metabolome and metabolomic networks can therefore potentially be highly informative [23].

We compared the metabolome in young, 0% cumulative mortality (CM) and middle-aged, 20% CM flies from C lines to flies of equal age from lines selected for increased mated longevity (CM’s of 0% and 8% respectively). We also sampled flies from the LS lines after 20% of the original cohort had died. If LS lines had a decreased rate of aging of the metabolome we expected a significant interaction between the effect of selection and aging.

## Results

**Figure 1:**
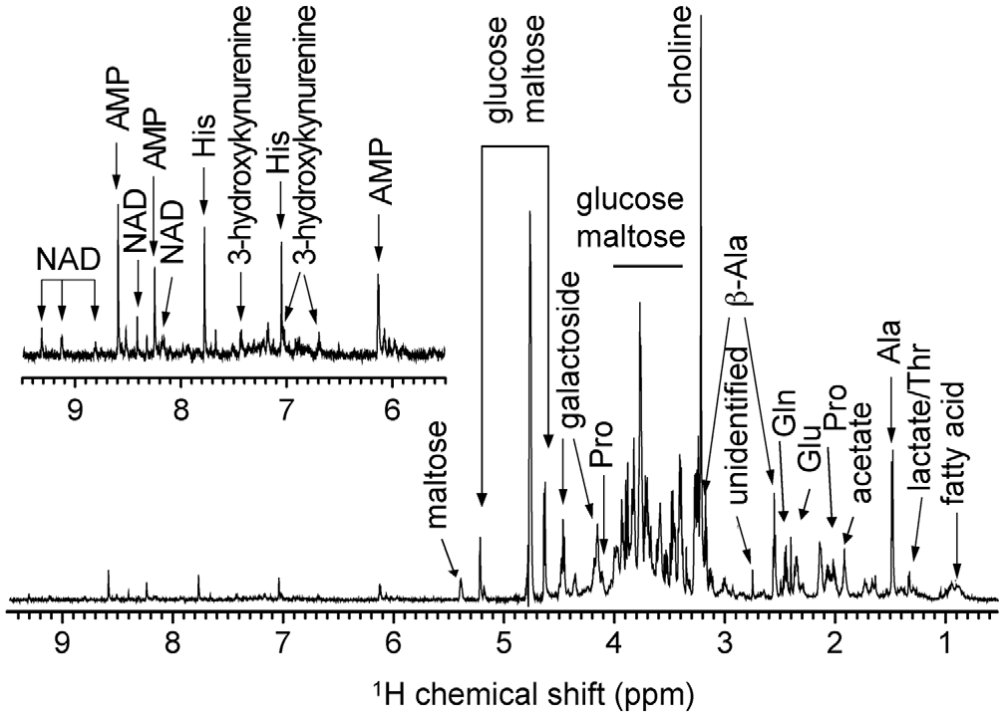
^1^H NMR spectrum of *Drosophila melanogaster* metabolites. CPMG spectrum of 3-day-old control flies acquired at 25°C and pH 7.4. Well-resolved signals are assigned. The scale in the insert is five times larger than in the full spectrum.

In this paper we present results from an NMR spectroscopic study of metabolomic changes as a function of age and selection for a long life in *D. melanogaster*. A typical *D. melanogaster* metabolite ^1^H NMR spectrum is shown in Fig. 1 where the well-resolved metabolite signals have been assigned.

**Figure 2:**
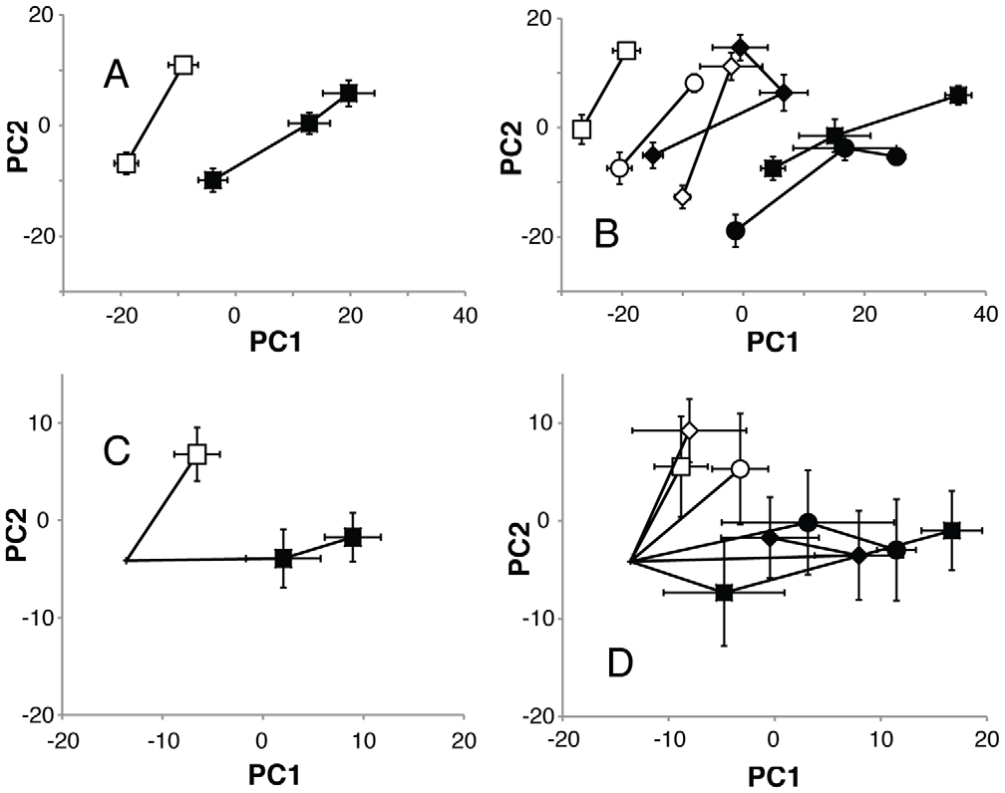
PCA scores for PC1 and PC2. PCA scores for PC1 and PC2 for C lines (open symbols) and LS lines (closed symbols) at day 3, day 19, and for LS lines day 35 with (A) showing selection regimes and (B) individual lines. The difference between old (day 19 and 35) and young (day 3) flies in the metabolome of selection regimes and individual lines are shown in (C) and (D), respectively. PC1 and PC2 explain 37 and 12% of the variation in (A) and (B), and 18 and 13% in (C) and (D). Error bars represent one standard error.

**Figure 3:**
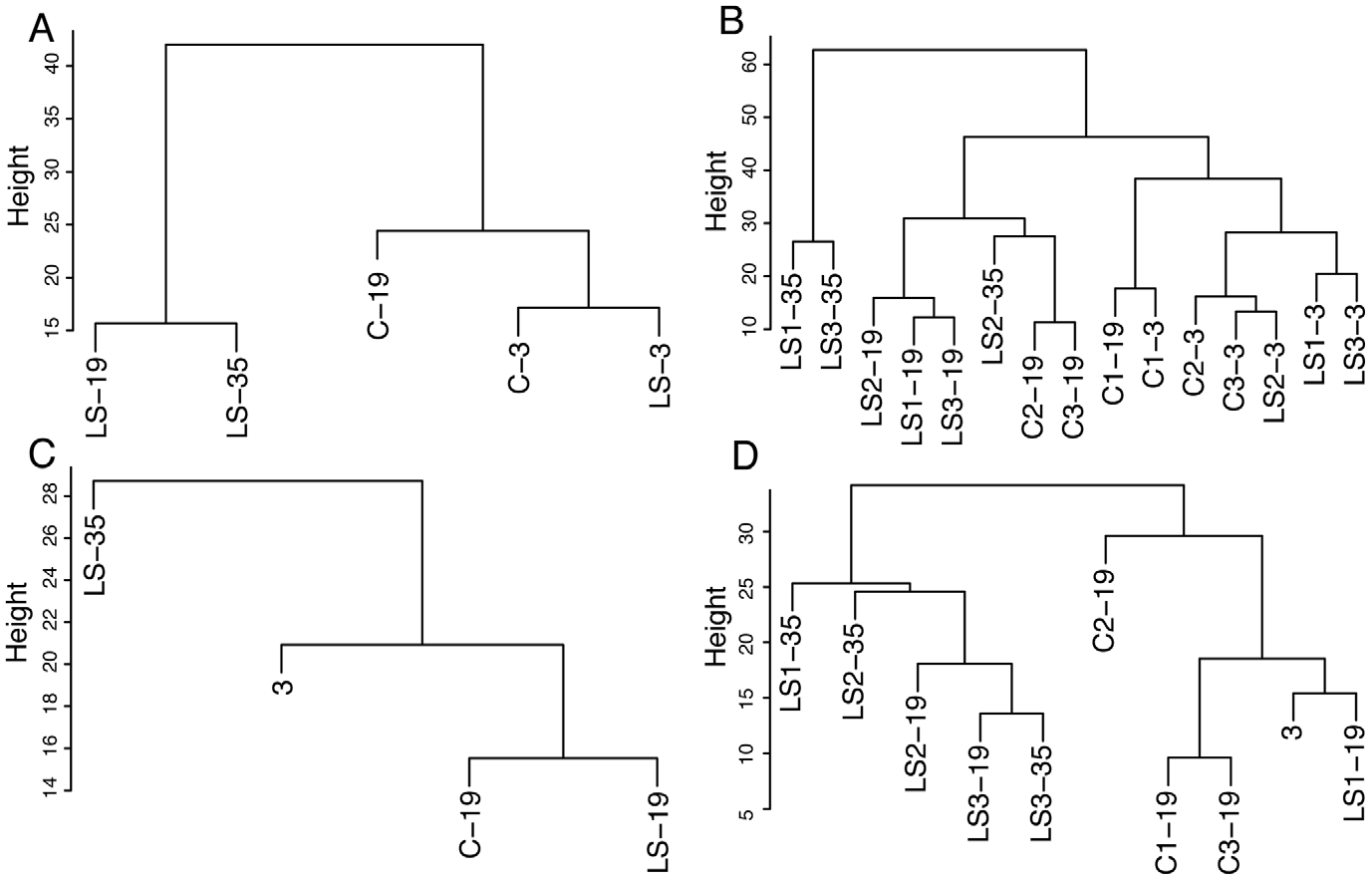
Hierarchical cluster analysis of the metabolic profiles. Hierarchical cluster analysis of the metabolic profiles of C and LS lines at day 3, day 19 and for LS lines at day 35 with (A) showing selection regimes and (B) showing individual lines. of the difference between old (day 19 and 35) and young (day 3) flies in the metabolome of selection regimes (C) and individual lines (D).

Five replicates from each of the three LS lines and three C lines were sampled at day 3 and 19 post eclosion and for LS lines also at day 35. We characterized the relative effects of age and selection for a long life by principal component analysis (PCA; Figs. 2A and 2B) and hierarchical cluster analysis on the full dataset (HCA; Figs. 3A and 3B). The PCA resulted in 7 principal components (PCs), which explained 71% of the variation and were tested for significant effects of age, selection and their interaction by a two-way-MANOVA (Tab. 1), showing significant effects of age and of selection for a long life. However, the interaction between age and selection for a long life was not significant in this analysis.

As seen in Figs. 2B and 3B the metabolomes of the different lines within selection regimes appear at quite different positions at day 3, a variation that is related to genetic variation among the genetically independent lines. In order to study the changes induced by aging undisturbed by the difference in metabolome induced by genetic background, we looked at the metabolomic changes from 3 to 19 days of age for the C lines and from 3 to 19 and 3 to 35 days for the LS lines. In this case PCA of a dataset containing the differences between all 19 or 35-day spectra and all 3-day spectra for each line resulted in 8 PCs, which explained 61% of the variation (Figs. 2C and 2D). A two-way-MANOVA (Tab. 1) showed significant effects of selection for a long life on the aging response between 3 and 19 days, implying that by looking at the changes relative to the 3-day flies we can indeed detect a difference in the aging response due to selection. It also showed that the LS flies have changed further after 35 days of age. The angle between overall score change between 3 and 19 day old flies for C and LS flies is approximately 45°.

**Table 1:** Two-way MANOVA of PCA scores based on signal intensities or changes^a^ in signal intensity.

Analysis	P (selection)	P (age)	P (interaction)
Intensities	6×10^−26^***	5×10^−35^***	0.2
Intensity changes^a^	2×10^−15^***	1×10^−38^***	n/a

* Significance (P = 0.05, 0.01, 0.001).

a relative to young (day 3) flies.

In contrast to the score plots that only account for the variation along the first two principal components, the dendrograms summarize all the variation in the dataset. As shown in Fig. 3A, the C and LS lines are relatively similar after 3 days. After 19 days the C flies cluster close to the 3-day flies while the LS-19 flies form a separate cluster together with the LS-35 flies. This pattern is not maintained when looking at the age-induced changes (Fig. 3C), where the C-19 and LS-19 flies cluster together, while the LS-35 flies are the most different from the rest. Similar patterns appear when looking at the individual lines (Figs. 3B and 3D), in this case however, the clustering of the patterns found for absolute levels and induced changes are switched so that Fig. 3B is more similar to 3C and Fig. 3D is more similar to Fig. 3A.

To characterize the relative effects of age and selection the average total score difference (PC1-7) between all samples within 3 and 19 day old C and LS flies were calculated. The age-induced change is 10% larger in LS flies and the difference between LS and C is 19% larger at 19 than at 3 days.

The metabolites that are different in young LS flies compared to young C flies and those that are affected by the ageing process in LS and C lines were identified using OPLS-DA on the metabolite NMR spectra. The difference between the aging processes for LS and C flies was determined by OPLS-DA from the difference data described above (Tab. 2). The metabolites that are affected by age, life expectancy and death rate (using previously obtained data, survival curves can be seen in figure S1) were identified using OPLS including all the samples in the dataset. The metabolites that change according to OPLS models are described in Tab. 3. All comparisons between C and LS flies at different ages, and the general model for age gave good predictions (high *Q* ^2^), while the prediction of the interaction term and of life expectancy and death rate were less good, but still useful (Tab. 2). It should be mentioned that the predictability *Q* ^2^ values were calculated using a cross-validation procedure where all the data for one combination of line and age was left out at a time. Regular cross-validation excluding random samples gave higher *Q* ^2^ values and a lower number of model components (*A*). The metabolites in Tab. 3 are those that show a high correlation with the predictive scores, before (*R* ^2^>0.35) or after (*R* ^2^>0.5) removing the variation explained by the orthogonal components in the model. The cutoffs were chosen because at this level most of the metabolites identified by multiple t-tests at the 0.05 level were included (not shown). While both procedures identify the metabolites that have a high correlation with the factor modeled for, the second one also includes metabolites that have a high correlation only after removing line dependent offsets accounted for by the model.

**Table 2:** Summary of OPLS-DA and OPLS models.

Comparison/Characteristic	*A* ‡	*n* §	R^2^X*	Q^2^ **
LS-3– C-3	1+5	29	0.61	0.93
C-19– C-3	1+5	29	0.60	0.96
LS-19– LS-3	1+6	29	0.68	0.95
LS-35– LS-19	1+6	29	0.72	0.96
LS-age interaction***	1+5	140	0.53	0.86
Age	1+4	72	0.47	0.95
Life expectancy	1+7	72	0.64	0.77
Death rate	1+7	72	0.63	0.60

* *R* ^2^ *X* describes how much of the total variation is explained by the model.

** *Q* ^2^ represents the predictability of the total model and is related to the statistical validity of the model. OPLS-DA showing Q^2^ values >0.5 is considered significant. *Q^2^* was calculated using cross-validation with all measurements for one line at one age left out one at a time.

‡ A describes the number of model components.

§ n describes the number of observation in the model.

*** Based on differences between 3 and 19 day old flies.

**Table 3:** Metabolite changes/differences with time and/or selection regime or viability characteristics as determined by OPLS-DA or OPLS.

Metabolite*	^1^H chemical shifts (ppm)	LS-3 C-3	C19 C-3	LS-19 LS-3	LS-35 LS-19	LS-3->LS-19C-3->C-19	Age	Life expectancy	Death rate
AMP	8.6, 6.13, 4.35		−**	**−**			**−**		
3-hydroxykynurenine	7.44, 6.98, 6.69,4.13, 3.18				**−** O		**−** O		
Maltose	5.4, 5.21, 4.64	**+**	**+** O	**+** O			**+** O	**+** O	**−** O
Glucose	5.21, 4.64	**+** O	**−** O					**+** O	
1-O-(4-O-(2-aminoethyl phosphate)-β-d-galactopyranosyl)-x-glycerol	4.48, 4.18, 3.93, 3.78, 3.26	**−**	**+**					**−**	**+**
Choline	3.18	**−** O	**−**	**−**		**+** O	**−**		
β-alanine	3.17, 2.54					**−**			**+**
Lysine	3.00, 1.90, 1.70	**−** O	**−** O	**−** O		**−** O	**−** O		
Glutamine	2.42, 2.12	**−**	**−**	**−**	**−**		**−**		
Alanine	1.45		**−** O					**+** O	
Lactate/Threonine	1.30		**+**	**+** O					
Fatty acid	0.91, 0.88	**−** O	**+**	**+** O				**−**	**+**
Unidentified metabolite	3.03, 2.74, 2.31, 1.90	**−**	**−**	**−**			**−**		

* - indicates a lower level in the upper category, or a decrease with increasing value of the life history parameter, while + indicates a higher level in the upper category, or an increase with increasing value of the life history parameter.

O the difference was only significant after removing the orthogonal variation explained by the model.

### Network Analysis

The processed dataset was subjected to network analysis using Weighted Gene Co-expression Network Analysis (WGCNA [24]) to identify modules which show an effect of selection, age or an interaction between the two on the bin intensities. We identified four modules and 64 bins that were not included in any of them. The eigenmetabolite summarizes the intensity of each module. It is the first principal component of a PCA on the bin intensities of a particular module and was used to detect whether the modules were associated with ageing and selection (Tab. 4).

**Table 4:** Results from two-way ANOVA analysis of eigenmetabolites. The number in parenthesis denotes the number of bins per module.

	source	DF*	MS**	F	P
Module 1	Selection	1	0.0434	4.2187	0.04383
(64)	Age	1	0.214378	20.8386	2.16E-05
	Sel x Age	1	0.042667	4.1474	0.0456
	Error	68	0.010288		
Module 2	Selection	1	0.34422	53.0789	4.33E-10
(332)	Age	1	0.21334	32.8978	2.45E-07
	Sel x Age	1	0.00146	0.2247	0.637
	Error	68	0.00871		
Module 3	Selection	1	0.33663	38.6531	3.54E-08
(142)	Age	1	0.01567	1.7997	0.18421
	Sel x Age	1	0.05548	6.3707	0.01394
	Error	68	0.59221		
Module 4	Selection	1	0.039825	2.9007	0.0931
(52)	Age	1	0.025237	1.8381	0.1797
	Sel x Age	1	0.001318	0.096	0.7576
	Error	68	0.01373		
Bins that does not belong to any module	Selection	1	0.034302	2.9799	0.0888465
(66)	Age	1	0.180576	15.6869	0.0001815
	Sel x Age	1	0.002357	0.2048	0.6523374
	Error	68	0.011511		

* degrees of freedom.

** mean squares.

### Dry Weight and Relative Fat, Nitrogen and Fat Free Carbon Content

**Figure 4:**
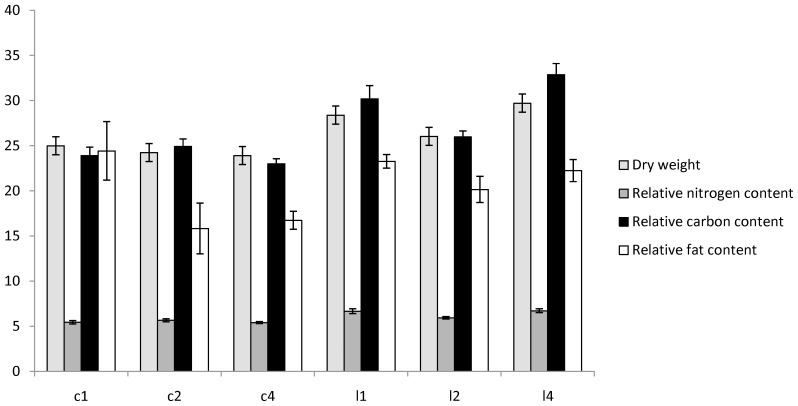
Body composition. Dry weight in 100 mg, relative nitrogen, carbon, and fat content (% of dry weight) in LS and C lines with standard deviations.

We found significantly higher dry weight and higher relative nitrogen and carbon content in the LS lines compared to C lines (Tab. 5, Fig. 4). There was no difference in relative fat content.

**Table 5:** Results from one-way ANOVA analysis of dry weight and relative fat content of males, with line nested within selection regime.

	Source	DF*	F Ratio	p
Dry weight	selection	1	10.44	0.03
Relative nitrogen content	selection	1	12.09	0.02
Relative carbon content	selection	1	7.24	0.05
Relative fat content	selection	1	1.09	0.36

* degrees of freedom.

## Discussion

As could be expected the results clearly show that both ageing and selection for increased longevity affect the metabolome of *D. melanogaster*. A clear separation in metabolic profile according to lifespan expectancy has also been found in *C. elegans* [25], [26]. We expected the longevity selected lines to have a younger metabolomic profile compared to same aged C flies, as had been found for the transcriptome [14]. However, cluster analysis (Fig. 3A) shows that while the metabolome of the LS flies ages differently from C flies it is not more similar to the metabolome in young flies. Actually, opposite to expectations, the metabolome of ageing LS flies is more different from that found in young flies in both selection regimes. The lack of concordance between the overall patterns found in the transcriptome and the metabolome, can be explained by modifications at the biological levels that lie between gene expression and the metabolome.

When the genetic differences in the young metabolome are removed from the cluster analysis of the age related changes in the metabolome they show that the metabolome of LS-19 flies resembles 19 days old C flies more than they do LS-35 flies (Fig. 3C). Nevertheless, the LS lines become increasingly different in the metabolome from both the C lines and the young LS lines as they age. There could be two distinct reasons for this. The metabolome of the LS flies could be ageing at a faster rate than that of the C lines, changing essentially the same metabolites in the same direction as C lines. Or the metabolome of LS lines could age differently from C lines, changing relative concentrations of metabolites that are not affected by age in C lines or changing the same metabolites in a different direction. As shown in Figs. 2C and 2D the vectors that describe the age related change in LS flies are not parallel with the corresponding C vector (resulting in a significant effect of selection for the difference data in Tab. 1), and the angle between overall change is approximately 45°. The LS metabolome thus experiences a different change with age. This difference is described in table 3 and discussed below. The influence of selection on the ageing of the metabolome was also evident in the results of the network analysis (Tab. 4) where 31% of the bins included in the analysis belonged to one of the modules displaying a significant interaction between the effects of selection and ageing. OPLS models were also created for age, survival and life expectancy (Tab. 2). It should be noticed that the model for age was by far the best both in terms of *Q* ^2^ value and a low number of fitted components. This indicates that even though selection has increased the average lifespan of LS lines chronological age is still the best predictor of the properties of the metabolome.

In addition to the difference in the metabolome between the selection regimes, the different genetic backgrounds of the lines within selection regimes had a clear influence on the metabolomic profile (Fig. 2B). These line differences were consistent with age, which indicates that they were not due to experimental noise, but rather to the independent laboratory evolution of the lines through more than 80 generations. Genetic drift is always a factor in evolutionary analyses, and the variation among lines within each selection regime in this experiment highlights the need for replicate lines in these studies.

### Metabolites Affected by Selection

As discussed above Tab. 3 shows metabolites that are highly correlated with the property modeled for both before and after removing the variation explained by the model. The second procedure allows for the identification of metabolites that would otherwise be hidden by line dependent offsets. Eight metabolites were affected by selection, two sugars (maltose and glucose) with higher relative concentration in LS lines, and a galactoside (1-O-(4-O-(2-aminoethyl phosphate)-β-d-galactopyranosyl)-x-glycerol), two lipids (choline and fatty acid), two amino acids (glutamine and lysine) and an unidentified metabolite with lower relative concentrations in LS lines (Tab. 3). The galactoside is synthesized exclusively in the accessory glands in males of some *Drosophila* species [27]. It has been found to have a lower relative concentration in inbred flies [15]. Lower concentrations of the galactoside and similar agents in inbred flies might have a negative effect on reproduction which is a trait normally suffering from inbreeding depression. There have been a lot of results indicating a trade off between early reproduction and longevity [28], [29]. However, lately studies have shown that the connection between reproduction and lifespan can be uncoupled [30] and there is no difference in the reproductive capacity of LS and C line males (Janneke Wit personal communication). The unidentified metabolite contains a methyl group bound to a nitrogen or similar, like in sarcosine, and a -(CH_2_)_3_- group in an environment similar to γ-butyric acid (GABA). Though the proton chemical shifts of the -CH_3_ (2.74 ppm) and -(CH_2_)_3_- groups (3.03, 2.31 and 1.90 ppm) agree well with sarcosine and GABA, the carbon chemical shifts do not, and both the carbon connectivities and the statistical correlations suggest that these groups belong to the same molecule. The metabolites affected by selection were very similar to the metabolites that are differentially regulated in long lived *C. elegans* [26]. Fuchs *et al.* studied the metabolic profile of several long-lived *C*. *elegans* strains including mutants of daf2 and dauer larvae. They found that several metabolites were differentially regulated in most long-lived strains although not consistently increasing or decreasing in relative concentration with longevity. Among these metabolites were O-Phosphocholine (increased in three and decreased in four out of nine strains), choline (increased in seven out of nine strains), glucose (increased in two out of nine strains) and lysine (increased in six out of nine strains). These results point to the relative concentration of choline variants, glucose and lysine being important for longevity in organisms that are quite evolutionary distant. There is no immediate evident explanation as to why both decreasing and increasing relative concentrations of these four metabolites should be associated with longer lifespan. However, this inconsistency might reflect a more or less complex dose-response relationship where the result of decreasing the relative concentration of the metabolite depends on its initial concentration and potentially on the state of the metabolome as a whole.

The link between choline and longevity is also supported by a study on dietary restricted mice and two types of long-lived mutant mice [31], where the relative concentrations of choline were decreased in the long-lived mice compared to their respective controls. Although this study used blood plasma samples and we should be cautious comparing results between single tissue and whole body experiments, choline does look like a candidate for further ageing studies. In mammals choline metabolism has been linked to Alzheimer’s disease (AD) [32], [33]. The Drosophila genome contains a homologue to the human β-amyloid precursor protein (APP), however, the sequence of the principal component of the AD associated amyloid deposits is not conserved [34] and normal flies do not develop AD like symptoms [32]. Still, choline’s involvement in neuronal signaling and membrane function makes it a key player in the normal functions of organisms and therefore a potential potent target for the age-related deterioration associated with senescence.

Increased relative abundance of maltose has been linked to temperature stress and inbreeding, presumably because it can protect membranes and proteins [15], [20], [35], [36], and these properties might also contribute to the increased lifespan in LS lines. Positive genetic correlations between stress resistance and longevity have frequently been reported [37], and maltose might be one possible link between the traits. However, contrary of what could be expected from the increased relative abundance of maltose in LS lines, the LS lines are not more heat and cold resistant than the C lines (Janneke Wit personal communication).

### Metabolites Affected by Age

Eleven metabolites were significantly affected by age in the control group. Eight of these were also significantly affected in the longevity-selected group and no other significant metabolite changes with age were found in this group (Tab. 3). The corresponding numbers of significantly affected metabolites when only accounting for those metabolites that showed a direct correlation with the predictive scores were 7 and 4, respectively. The reason for the difference in the results was likely due to the larger spread in metabolite profile between LS compared to C lines. Significant differences in the aging process are discussed below. The higher agreement when including also metabolites accounted for by the full model shows the strength of this procedure. Eight of these metabolites also differed in relative concentration among selection regimes at day 3 giving further indications to these metabolites being involved in both the processes of ageing and lifespan determination. Five of these were changing in the same direction due to longevity selection as with age.

The increase with age in the relative abundance of lactate and decrease in AMP in both selection regimes supports the well founded hypothesis that energy metabolism is changing as organisms age [38]–[40]. However, we have no evidence of energy metabolism being the target for selection in the LS lines, as none of these metabolites are significantly different in relative abundance or ageing response between selection regimes (Tab. 3).

Three un-branched amino acids or metabolites of amino acids (lysine, glutamine and 3-hydroxykynurenine) and the unidentified metabolite had low relative concentrations in LS lines as well as decreasing relative concentrations with age in both C and LS lines. The low abundance of free amino acids in LS lines is not reflected in a low nitrogen content over all, just as the low relative concentration of fatty acids in LS lines does not result in a low relative fat content (Fig. 4). These four metabolites also react to a cold shock in *D. melanogaster* [20]. 3-hydroxykynurenine decreases and the other three amino acids increase in relative concentrations following a sharp 30**°**C drop in ambient temperature. The low initial concentration of these metabolites in LS lines might contribute to their low cold resistance compared to C lines.

We found lower relative concentrations of 3-hydroxykyrunine in LS35 compared to LS19. This metabolite has previously been shown to have a lower concentration in inbreed lines and following a cold shock in Drosophila [15], [20]. In addition, 3-hydroxykynurenine is a signal of oxidative stress and a neurotoxin [41], [42] and high levels of this metabolite are found in relation to many neurodegenerative disorders such as Parkinson’s, Alzheimer’s and Huntington’s disease [43], [44] and have been linked to increased levels of reactive oxygen species [41].

### Metabolites Affected Differently by Age in Longevity Selected Flies

In the longevity selected flies the choline variant decreased more with age while lysine decreased less. There was also a relative decrease in β-alanine in longevity-selected flies relative to control flies. It is not likely that these three metabolites alone, can explain the clear differences in ageing effects between LS and C lines seen in Figs. 2C and 2D and Table 4. It is the result of comparably small changes in the relative concentration of many different metabolites, most of which were not individually significant. This is also indicated by a high number of metabolites that have a relatively high loading on the PC’s depicted in Figs. 2C and 2D.

In conclusion we found that the metabolome of long lived lines differ from the control both at young age and in their response to ageing. This does not result in a younger metabolic profile of LS lines but rather in a different metabolic profile in LS lines. Of the eight metabolites that differed in relative abundance between selection regimes choline is especially interesting as it has now been associated with variation in lifespan phenotypes in very evolutionary distant lineages (arthropods, nematodes and mammals). The mechanism though which the relative concentration of choline affects lifespan should be investigated.

## Experimental Procedures

### Origin of Flies and Selection Regime

The replicate selection and control lines originate from a mass population of flies from mixed geographic origin. The origin of flies and the set up of the mass population is described in more detail in [45]. The mass population was maintained as one interbreeding population for four generations before three replicate selection and control lines were established. Each replicate line was maintained in 5 culture bottles with a minimum population size of 60 pairs in each (in total a population size of 300 pairs). The 5 bottles, within a replicate line, were mixed each generation to reduce the effect of drift. The longevity selection took place every other generation, where flies after emergence were placed in food vials and transferred to new ones every second day until approximately 50% of the flies are dead. At the first generation of selection this took four weeks, after 41 generations of selection 50% mortality was reached after approximately 6½ weeks. The surviving flies were allowed to start the next generation. Replicate lines of the control regime were kept at standard laboratory conditions at 25°C and a 12/12 h light/dark cycle on standard agar-sugar-yeast-oatmeal medium. The flies used for experimentation were offspring from an unselected generation to avoid any cross-generational effects of the selection procedure [46]–[48]. For a more detailed description of selection regimes see [14].

### Sampling

Seven day old Flies were set-up in 200 ml bottles with 35 ml standard medium under uncrowded conditions (allowing 10 pairs to lay eggs for 24 h per bottle), upon the start of emergence the bottles were emptied and flies were collected less than 12 h old under light CO_2_ anesthesia. We collected 15 males and females per vial and 14 vials from each replicate line. The flies were transferred to new food vials, with 4 ml standard Drosophila oatmeal-sugar-yeast-agar medium containing antibiotics (alternating between Ampicillin 0.1g/L and Doxycycline 0.25 g/L), every second day, and the number of vials was gradually reduced as deaths occurred, with surviving flies being kept at a density as close to 30 as possible. The day before sampling the sexes were separated under light CO_2_ anesthesia and from each combination of line and age we took 5 samples containing 50 males each. Samples were frozen in liquid nitrogen at the same time of day (3 p.m.), to prevent circadian rhythm from disturbing the results, on day 3, 19 and for LS lines day 35 after collection. Average cumulative mortalities in the LS lines at the 3 time points were: LS3∶0% and LS19∶8% and LS35∶20% respectively, while in the 3 C lines it were C3∶0% and C19∶20%.

### Preparation of Samples and ^1^H-NMR Analysis

Flies from each sample were mechanically homogenized with a Kinematica, Pt 1200 (Buch & Holm A/S) in 1 mL of ice-cold acetonitril (50%) and centrifuged for 30 min (4°C). The supernatant was transferred to new tubes, lyophilized and stored at −80°C. Immediately before the NMR measurements, samples were rehydrated in 650 µL of 50 mM phosphate buffer (pH 7.4) in D_2_O, and 600 µL was transferred to a 5 mm NMR tube. The buffer contained 50 mg/L of the chemical shift reference 3-(trimethylsilyl)-propionic acid-D4, sodium salt (TSP). The NMR measurements were performed at 25°C on a Bruker Avance-2 700 spectrometer (Bruker Biospin, Rheinstetten, Germany), operating at a ^1^H frequency of 700.09 MHz, equipped with a 5 mm HCN triple resonance probe. ^1^H NMR spectra were acquired using a single-90°-pulse experiment with a Carr-Purcell-Meiboom-Gill (CPMG) delay added in order to attenuate broad signals from high-molecular-weight components. The total CPMG delay was 40 ms and the spin-echo delay was 200 µs. The water signal was suppressed by presaturation of the water peak during the relaxation delay of 1.5 s. A total of 256 transients of 16 K data points spanning a spectral width of 24 ppm were collected, corresponding to a total experimental time of 10 min. For assignment purposes a 2-dimensional ^1^H-^1^H COSY, ^1^H-^1^H TOCSY, ^1^H-^13^C HSQC, ^1^H-^13^C HSQC-TOCSY and a ^1^H-^13^C HMBC spectrum with suppression of one-bond correlations were acquired (Figure S2). 3-hydroxykynurenine was identified using spiking in an earlier study [15]. The other metaboliltes were identified from ^1^H and ^13^C chemical shifts only.

### Data Reduction and Statistics

The spectra were processed using iNMR (www.inmr.net). An exponential line-broadening of 0.5 Hz was applied to the free-induction decay prior to Fourier transformation. All spectra were referenced to the TSP signal at −0.017 ppm, automatically phased and baseline corrected. The spectra were aligned using icoshift [49]. Data reduction was accomplished by dividing the spectrum into 0.01 ppm regions (bins) over which the signal was integrated to obtain the signal intensity. The region around the residual water signal (4.85–4.7 ppm) was removed in order not to compromise the analysis. The high- and low-field ends of the spectrum, where no signals except the reference signal from TSP appear, were also removed (i.e. leaving data between 9.5 and 0.5 ppm). The integrals were normalized to total intensity in order to suppress trivial separation based on variations in amount of sample.

Principal component analysis (PCA) was carried out on the full data. Data was scaled using VAST scaling [50] to obtain unit variance within each line at each age on average (i.e. each region/bin was divided by the average standard deviation of the integral of that region for all combinations of line and age) and then centered. This scaling reduces the weight of variations that are not related to the treatments, i.e. random variations between “identical” samples are reduced, and the analysis is not biased towards metabolites present at high concentrations. Initial PCA identified one replicate of a longevity line, age 35 days as a significant outlier. This replicate was excluded. The number of significant PCs was assessed by leave one out cross validation [51], leaving out all samples for each combination of line and age at a time. The scores from the PCA were subjected to two-way ANOVA for line and age. OPLS discriminant analysis (OPLS-DA) [52] was carried out between control and longevity flies at all ages to identify metabolites affected by age and by selection for longevity. Orthogonal projection to latent structures, OPLS, separates the variance in x correlated with y (y-predictive) with the orthogonal (non-correlated; y-orthogonal) variance [53]. In contrast to regular PLS, a single y will result in only one predictive component. OPLS-DA was performed on UV-scaled data as this gave the best separation between classes. To facilitate interpretation of OPLS-DA loadings were back transformed with the UV scaling factor and for each bin the correlation coefficient (*R*) between comparison and the predictive component after removing the variation explained by the orthogonal component was calculated. Metabolites showing R^2^>0.5 were considered significant. This cutoff was chosen since the metabolites identified this way included those that were identified by multiple t-tests at the 0.05 level applying Bonferroni correction for an assumed number of 100 metabolites in the sample. The metabolites affected by age, life expectancy (calculated as the average remaining life of the flies alive) and death rate (calculated as number of dead flies the next day divided by the number of living flies) were similarly identified using OPLS including all the samples in the dataset. Multivariate data analysis was performed using Simca P+12.0 (Umetrics, Umeå). Analyses of the changes relative to the 3-day samples were based on the differences in peak intensities between all older and 3-day samples from the same line. The significance levels were calculated based on the degrees of freedom of the original unexpanded data set.

### WGCNA Network Analysis

The structure of the dataset was explored using weighted gene co-expression network analysis (WGCNA for R). This analysis constructs a adjacency matrix based on correlations between metabolites weighted by the unsigned correlation coefficient [24]. For each combination of sample and module the “eigenmetabolite” (called eigengene by WGCNA) was calculated. The eigenmetabolite is the first principal component resulting from a PCA analysis on the intencities of all the metabolites included in a given module, and was used to establish the effect of selection and ageing on the module through a two-way ANOVA.

### Dry Weight and Relative Fat, Nitrogen and Fat Free Carbon Content

Eggs were collected and allowed to develop in vials containing 7 ml standard medium at a density of 20 per vial. Upon the start of emergence the vials were emptied and flies were collected and sexed less than 8 h old under light CO_2_ anesthesia. The flies were aged six days at a density of 30 pr vial with equal sex ratio in food vials. New vials were provided every second day. At day 6 post eclosure samples of 30 males from each replicate line were snap frozen in liquid nitrogen. We used the method described by Fairbank and Burch [54] to determine dry weight and ether-extractable fat content. The flies were dried in a vacuum oven at 60°C for 48 h in open Eppendorf vials before being weighed individually to the nearest 0.001 mg. 2 ml of petroleum ether was added to each vial. The samples were kept at room temperature for 5 days. The flies were dried again at 60°C for 24 h after the petroleum ether was discarded, and subsequently weighed to obtain the fat free dry weight. Relative fat content (RFC) was calculated using
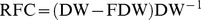
with DW = dry weight and FDW = fat free dry weight. The nitrogen content was measured using a dry combustion analyzer (Na2000, Carlo Erba, Italy). The data was analyzed by a one-way nested ANOVA with line nested within selection regime.

## Supporting Information

Figure S1 **Survival curves of mated males (14) open symbols: control lines, closed symbols longevity lines.**
(PDF)

Figure S2 A. Downfield region of TOCSY spectrum of three day old fruitflies selected for longevity. B. Upfield region of TOCSY spectrum of three day old fruitflies selected for longevity.
(DOCX)
