# Supplementary figures and images for: The Metabolic Profile of Long-Lived Drosophila melanogaster
Source: PLoS One. 2012 Oct 23;7(10):e47461. doi: 10.1371/journal.pone.0047461 (PMC3479100; doi:10.1371/journal.pone.0047461)

Survival curves of mated males (14) open symbols: control lines, closed symbols longevity lines

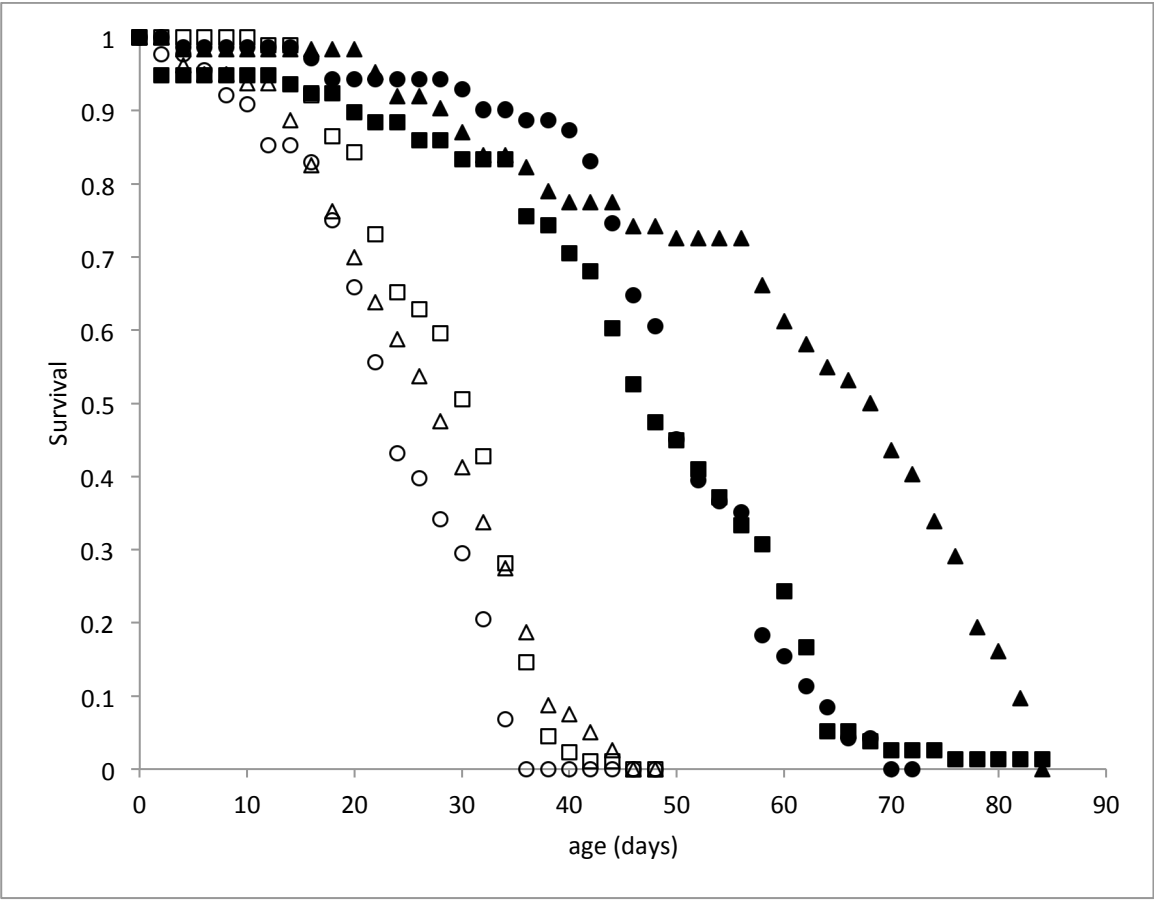

Supplement: Figure S1 — Survival curves of mated males (14) open symbols: control lines, closed symbols longevity lines. (PDF) [file pone.0047461.s001.pdf]
