# Supplementary material for: The Metabolic Profile of Long-Lived Drosophila melanogaster
Source: PLoS One. 2012 Oct 23;7(10):e47461. doi: 10.1371/journal.pone.0047461 (PMC3479100; doi:10.1371/journal.pone.0047461)

*Supplementary Material*

The metabolic profile of long-lived *Drosophila melanogaster*

Pernille Sarup, Simon Metz Mariendal Pedersen, Niels Chr. Nielsen,
Anders Malmendal, Volker Loeschcke

Figure S1A. Downfield region of TOCSY spectrum of three day old fruitflies selected for longevity.


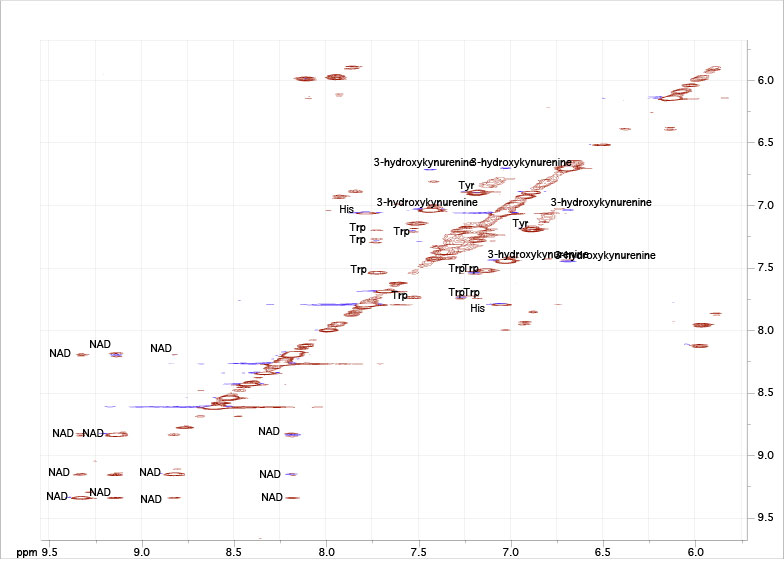


Figure S1B. Upfield region of TOCSY spectrum of three day old fruitflies selected for longevity.


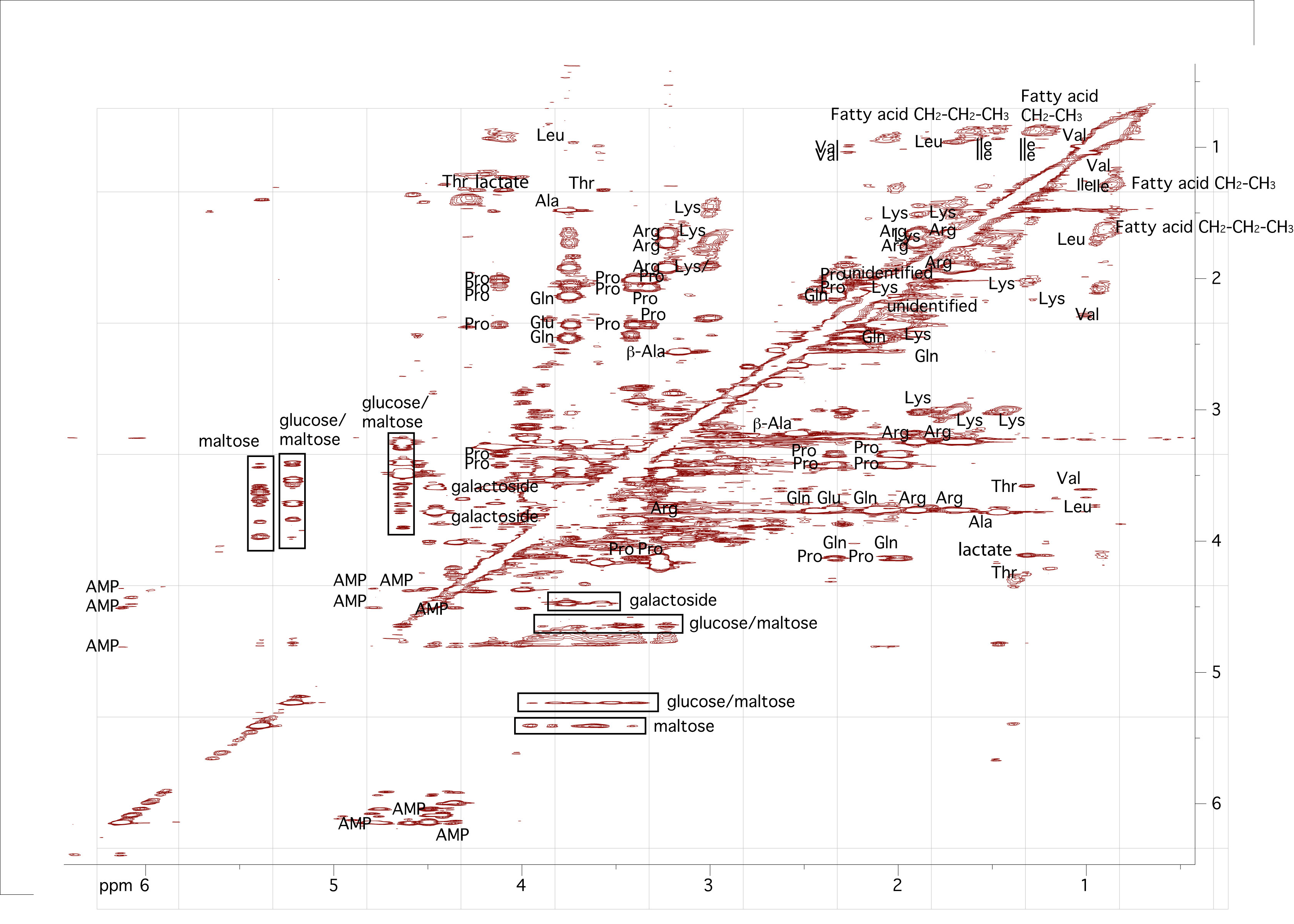

Supplement: Figure S2 — A. Downfield region of TOCSY spectrum of three day old fruitflies selected for longevity. B. Upfield region of TOCSY spectrum of three day old fruitflies selected for longevity. (DOCX) [file pone.0047461.s002.docx]
